# Task-Specific Reliability and Measurement Error of Frontal Plane Kinematics in Individuals with Patellofemoral Pain: A Preliminary Study

**DOI:** 10.3390/mps9030076

**Published:** 2026-05-13

**Authors:** Hiraku Nagahori, Isabella Keefer, Derrick Smith, Brendan Yawn, Jing Nong Liang, Kai-Yu Ho

**Affiliations:** Department of Physical Therapy, University of Nevada, 4505 S. Maryland Pkwy, Las Vegas, NV 89154, USA; nagahh1@unlv.nevada.edu (H.N.); keefer@unlv.nevada.edu (I.K.); smithd89@unlv.nevada.edu (D.S.); yawn@unlv.nevada.edu (B.Y.); jingnong.liang@unlv.edu (J.N.L.)

**Keywords:** reliability, frontal plane, kinematics, minimal detectable change

## Abstract

This study evaluated the test–retest reliability, standard error of measurement (SEM), and minimal detectable change (MDC) of frontal plane projection angles (FPPAs) across five single-leg tasks in individuals with patellofemoral pain (PFP). Two-dimensional video data was collected from ten individuals with predominantly unilateral PFP. Participants performed single-leg squat, single-leg landing, single-leg hop, forward step-down, and lateral step-down across two testing sessions. FPPAs were measured at peak knee flexion for each task, including trunk lean angle, knee FPPA, hip FPPA, and dynamic valgus index. Test–retest reliability was assessed using intraclass correlation coefficients (ICCs). Our findings indicate that test–retest reliability and measurement error for trunk and lower limb FPPA varied across tasks in individuals with PFP. The lowest ICC was observed for hip FPPA, particularly during single-leg squat and lateral step-down tasks. Among the five tasks tested, the single-leg squat appeared to be the most demanding task, demonstrating the lowest ICCs, and highest SEM and MDC values across all four outcome measures (trunk lean angle, knee and hip FPPAs, and dynamic valgus index). The dynamic valgus index consistently showed larger SEM and MDC values than isolated hip or knee FPPAs, likely reflecting compounded measurement errors across segments. These findings provide preliminary insights, though confirmation in larger samples in persons with PFP is warranted.

## 1. Introduction

Patellofemoral pain (PFP) is a prevalent musculoskeletal condition characterized by anterior knee pain that is typically exacerbated by activities such as squatting, running, stair ascent and descent, and prolonged sitting [1]. PFP is frequently associated with altered movement patterns, including increased knee valgus, excessive pelvic drop, and ipsilateral trunk lean during weight-bearing tasks. These deviations are often attributed to deficits in neuromuscular control and weakness of the hip musculature, particularly the hip abductors and external rotators, which are essential for maintaining pelvic stability and lower extremity alignment [2]. Such movement alterations can contribute to abnormal loading of the patellofemoral joint and may perpetuate symptoms over time [3].

Two-dimensional video analysis is widely used in clinical settings to assess frontal plane projection angles (FPPAs) due to its cost-effectiveness, portability, and ease of implementation compared with three-dimensional motion capture systems [4]. FPPAs quantify segmental alignment in the frontal plane, providing insights into joint and trunk kinematics during functional tasks. Assessing test–retest reliability using intraclass correlation coefficients (ICCs), as well as quantifying standard error of measurement (SEM) and minimal detectable change (MDC), is essential for distinguishing true changes in movement patterns from measurement error [5]. Specifically, SEM quantifies the amount of measurement error inherent in a test, while MDC represents the smallest change that can be interpreted as a true difference beyond measurement error [5].

From a methodological perspective, several factors influence the reliability of FPPA measurements, including anatomical marker placement, camera positioning, task standardization, the number of trials, and inherent movement variability (i.e., natural differences in how an individual performs a movement across repetitions of the same task). For example, more dynamic, complex, or demanding tasks may provoke greater movement variability or pain in individuals with PFP. Movement variability can introduce measurement noise that reduces the reliability of repeated movements and limits the ability to detect meaningful changes [6]. Consequently, increased movement variability may reduce test–retest reliability, resulting in lower ICC values and higher measurement errors as reflected by SEM and MDC.

Evidence regarding movement variability in individuals with PFP remains inconsistent. Some studies report increased variability in knee adduction and valgus angles during running, suggesting impaired motor control [7], whereas others described more rigid, stereotyped movement patterns that may lead to repetitive stress on the patellofemoral joint [8]. This lack of consensus limits the interpretation of movement variability in PFP and highlights the need for studies that quantify its impact on reliability measurements. In particular, establishing test–retest reliability and the corresponding SEM and MDC values for trunk and lower extremity FPPAs across clinically relevant tasks is essential. For example, if a change in hip FPPA is observed during a single-leg squat following an intervention in a patient with PFP, the SEM and MDC can help clinicians determine whether this observed difference in hip FPPA reflects a true change in motor behavior or is attributable to measurement error.

Despite the widespread clinical use of two-dimensional video analysis, there is limited research on the reliability and measurement errors of FPPA measures in individuals with PFP. Most existing studies have focused on pain-free populations, limiting the applicability of their findings to clinical populations [4,9,10]. Furthermore, MDC values for composite metrics such as the dynamic valgus index, which integrates contributions from knee and hip FPPAs, have rarely been reported in PFP populations. Given that individuals with PFP may demonstrate altered and/or inconsistent FPPA of the trunk and the lower extremities due to pain and/or altered movement control, reliability and measurement error references established in pain-free populations may not be generalizable to this patient population.

Therefore, the purpose of this study was to examine test–retest reliability, as well as SEM and MDC, for FPPAs of the trunk, pelvis, and lower extremities in individuals with PFP. By providing population- and task-specific estimates, this study aims to support the interpretation of movement assessment, and inform clinical decision-making in individuals with PFP.

## 2. Materials and Methods

### 2.1. Study Design

This study employed an observational, repeated-measures design to evaluate the test–retest reliability of two-dimensional frontal plane kinematic variables of the trunk and lower extremity in individuals with PFP. Specifically, the study quantified ICCs, SEM, and MDC values for FPPA-based variables across five single-limb, weight-bearing tasks. Two testing sessions were conducted to assess test–retest reliability while minimizing potential influences of learning effects.

### 2.2. Participants

Ten participants (6 males, 4 females; mean age 28.2 ± 6.88 years; body mass index 26.83 ± 6.67 kg/m^2^) with predominantly unilateral PFP were included in this analysis. Participants were recruited from the Las Vegas metropolitan area between 2022 and 2023 [1]. Inclusion criteria were: (1) age between 18 and 45 years; (2) a clinical presentation of predominantly unilateral PFP lasting at least 3 months; and (3) observable knee valgus during a forward step-down task. Participants reporting bilateral PFP were eligible if one limb was consistently more symptomatic and selected as the testing limb [1].

Exclusion criteria included the absence of observable knee valgus during screening, a history of significant lower-extremity musculoskeletal injury, or knee surgery within the preceding 12 months. Prior to participation, all individuals were informed of the study purpose, procedures, and potential risks. Written informed consent was obtained from all participants in accordance with procedures approved by the Institutional Review Board of the University of Nevada, Las Vegas [1].

### 2.3. Screening

All participants underwent a standardized physical examination conducted by a licensed physical therapist to confirm the presence of PFP. The examination included soft tissue palpation around the peripatellar region and a patellar compression test to reproduce familiar symptoms consistent with PFP [1]. Participants then performed a forward step-down task while frontal plane knee motion was visually assessed. Individuals without PFP symptoms or without observable knee valgus during the task were excluded from further participation.

### 2.4. Tasks

Before testing on each day, participants rated their pain using a 10-cm visual analog scale (VAS). Two-dimensional video data were captured using a Sony CX405 Handycam^®^ (Sony, Tokyo, Japan), positioned 15 feet directly in front of each participant to maintain consistent camera perspective and measurement accuracy. Reflective anatomical markers were placed on the sternum, as well as bilateral anterior superior iliac spine (ASIS), mid-thigh, mid-patella, and mid-ankle to define segment reference lines for kinematic analysis [1]. The mid-thigh marker of the weight-bearing limb was used as an additional marker to assist the identification of the thigh segment, only in cases where the ASIS marker of the weight-bearing limb was not visible due to arm movements or trunk flexion (see Data Processing for more details).

Participants completed two testing sessions separated by a minimum of 14 days. A minimum 14-day interval was selected to minimize recall, familiarization, and short-term motor learning effects associated with repeated task performance while limiting the likelihood of substantial clinical or movement changes in participants with PFP. While shorter intervals (e.g., 2–7 days) are commonly used in reliability studies [11], the longer interval in the present study may better reflect clinical reassessment durations and reduce the influence of learning effects on repeated movement testing outcomes. During each session, participants performed five functional tasks on the testing (symptomatic) limb: single-leg squat, single-leg landing, single-leg hop, forward step-down, and lateral step-down.

For the single-leg squat, they stood on the affected leg, squatted to a knee flexion angle of 45°, and then returned to the starting position over a 3-s duration [1]. During the single-leg landing task, participants stood on a 30 cm step using their symptomatic leg, jumped forward to a mark 30 cm away, and maintained balance for 3 s after landing [1]. The single-leg hop required participants to hop forward as far as possible on their symptomatic leg and sustain their balance for 3 s upon landing [1]. For the forward step-down, participants stood on a 30 cm step with the symptomatic leg, gently tapped the heel of the opposite foot on the floor in front, and returned to the initial position over a 3-s interval [1]. Similarly, in the lateral step-down task, they tapped the heel of the opposite foot on the floor to the side before returning to the starting position within 3 s [1].

Each task was repeated three times, with a 1-min rest between tasks. During these tasks, two trained Doctor of Physical Therapy students positioned themselves on either side of the participant as spotters to ensure safety in the event of a loss of balance. Participants were also allowed to take additional breaks as needed to minimize fatigue and maintain consistent performance.

### 2.5. Data Processing

Frontal plane kinematic variables were analyzed using Kinovea software (ver.2023.1). Variables of interest included trunk lean angle, knee FPPA, hip FPPA, and the dynamic valgus index, all extracted at the point of peak knee flexion during each task [4]. Specifically, the videos were imported into the Kinovea software and the angles were measured at the frame where the observed knee flexion was greatest. All analyses were conducted by the same rater, a trained Doctor of Physical Therapy student, to ensure consistency across all trials. Additionally, the rater was blinded to the testing sessions.

**Figure 1:**
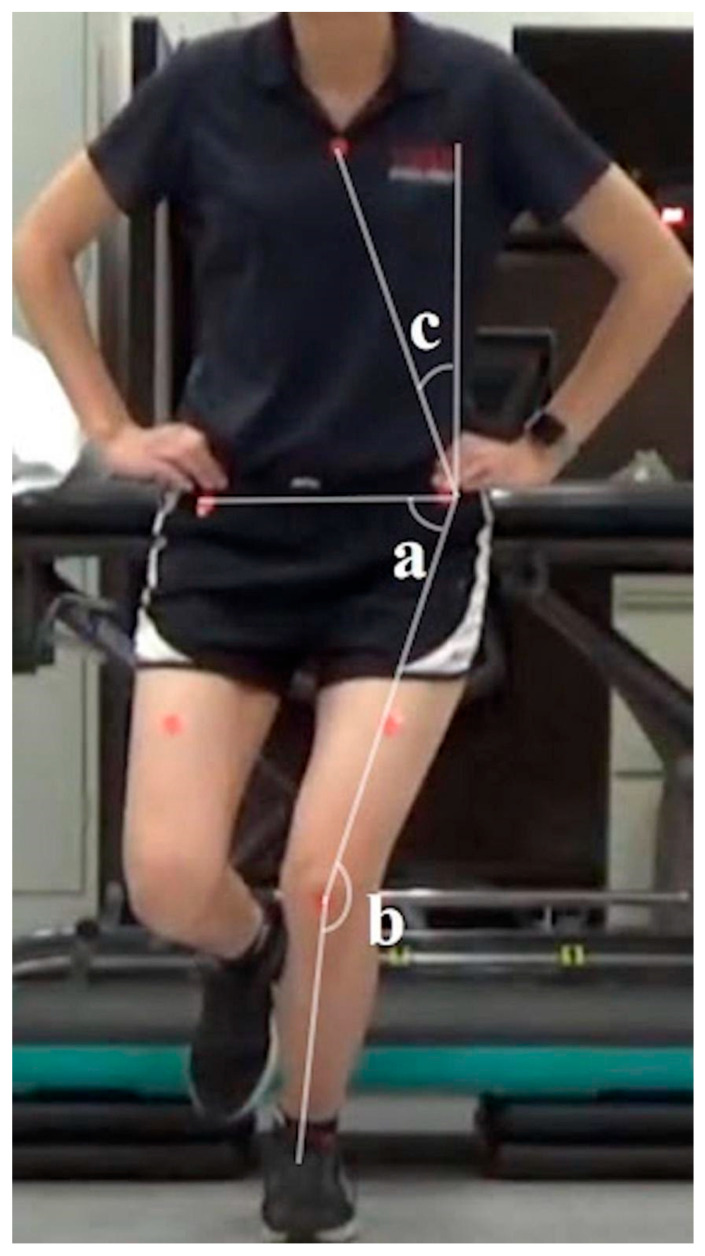
Example of two-dimensional frontal plane kinematics measured during a single-leg squat. Trunk lean angle = c; hip FPPA = 90 degrees—a; knee FPPA = 180 degrees—b; dynamic valgus index = hip FPPA + knee FPPA. Abbreviations: FPPA, frontal plane projection angle.

To calculate trunk lean angle, a vertical reference line was extended upward from the ipsilateral ASIS. A second line was drawn from the ASIS to the sternum marker, and the angle between these two lines was calculated (Figure 1). Lower trunk lean angle values indicated greater ipsilateral trunk lean [1]. Knee FPPA was calculated as 180° minus the obtuse angle formed between the thigh and shank segments, defined by a line from the ASIS to the mid-patella and a line from the mid-patella to the mid-ankle of the weight-bearing limb (Figure 1). Hip FPPA was calculated as 90° minus the angle between the pelvis and thigh segments, defined by a line connecting both ASIS and a line from the ASIS to the mid-patella of the weight-bearing limb (Figure 1). In our study, only ASIS markers were used to determine the hip FPPA (i.e., no mid-thigh markers were used), given that the ASIS markers across all 5 tasks were visible in all participants. Greater knee FPPA values indicate increased knee valgus, whereas higher hip FPPA values reflect greater hip adduction and/or contralateral pelvic drop [4]. The dynamic valgus index (°) was the sum of knee and hip FPPAs, capturing the combined effect of frontal plane kinematic deviations of the lower extremity (Figure 1) [1]. Each angle was averaged across three trials per task for analysis.

### 2.6. Statistical Analysis

Test–retest reliability was examined using ICCs (2-way random effects, absolute agreement, single measurement; ICC [2,1]) for each variable across the two testing days. ICC values were interpreted as poor (<0.4), fair (0.4–0.7), good (0.7–0.9), or excellent (>0.9) reliability [12]. SEM was calculated as SD × √(1–ICC), and MDC as SEM × 1.96 × √2 [4]. Relative SEM was determined by dividing the SEM by the mean frontal plane angle for each task, while relative MDC was calculated by dividing the MDC by the mean frontal plane angle for each task.

All analyses were performed for each of the five functional tasks: single-leg squat, single-leg landing, single-leg hop, forward step-down, and lateral step-down. Statistical analyses were conducted using Statistical Package for the Social Sciences (SPSS, version 23.0; IBM Corporation, Armonk, NY, USA). A significance level was established with a threshold of *p* ≤ 0.05.

## 3. Results

In this study, participants reported mean pain scores of 0.89 (SD = 1.24) on Day 1 and 1.33 (SD = 2.06) on Day 2 on the visual analog scale, with no significant difference observed between days (paired *t*-test, *p* = 0.461). Table 1 shows FPPAs across all five measured tasks. While the dynamic valgus index demonstrated the greatest values during the forward step-down task, the remaining FPPA measures demonstrated their greatest values during the lateral step-down task. Across all five tasks, knee FPPA values were generally smaller than the other FPPA measures.

Between-day test–retest reliability for frontal plane kinematic variables varied across tasks and measures (Table 1). Hip FPPA reliability ranged from poor to excellent, with the lowest ICCs observed during single-leg squat and lateral step-down tasks and the highest during the single-leg hop. Knee FPPA generally demonstrated fair to excellent reliability, with the highest ICC values observed in the forward and lateral step-down tasks. The dynamic valgus index showed good to excellent reliability across tasks, with slightly lower ICCs during single-leg squat and lateral step-down tasks. Trunk lean angle reliability ranged from fair to excellent, with the single-leg landing task showing the highest ICC value.

SEM and MDC values varied across measures, reflecting differences in measurement errors. Hip FPPA SEMs ranged from 1.70 to 3.87 degrees (MDC: 4.71–10.71°), while knee FPPA SEMs ranged from 1.66 to 4.29 degrees (MDC: 4.61–11.90°). The dynamic valgus index demonstrated higher SEMs (3.83–5.91°) and MDCs (10.61–16.37°), whereas the SEMs of trunk lean angle were lower (1.83–2.57°; MDC: 5.06–7.13°) (Table 1). Relative SEM and MDC values also varied across measures. Hip FPPA relative SEMs ranged from 0.14 to 0.32, with relative MDCs ranging from 0.39 to 0.89. Knee FPPA relative SEMs ranged from 0.18 to 1.27, while relative MDCs ranged from 0.50 to 3.53. The dynamic valgus index demonstrated relative SEMs ranging from 0.13 to 0.41 and relative MDCs ranging from 0.36 to 1.13. Trunk lean angle demonstrated lower relative SEMs (0.15–0.21) and relative MDCs (0.42–0.59) across tasks (Table 1).

## 4. Discussion

The purpose of this study was to evaluate test–retest reliability, as measured by ICC, and measurement error, as reflected by SEM and MDC, of FPPA measures of the trunk, pelvis, and lower extremities during commonly used single-limb weight-bearing tasks in individuals with PFP. This study provides important, task-specific reliability data and measurement error estimates for FPPA measures in individuals with PFP, addressing a notable gap in the current literature. These findings offer a foundation for interpreting movement assessments in this population.

Our findings showed that ICC, SEM, and MDC were task- and variable-specific, with some measures showing lower levels of ICC and greater SEMs and MDC than others. The lowest reliability was observed for hip FPPA during the single-leg squat (ICC = 0.396, MDC = 8.61°), indicating poor reliability. Variables demonstrating fair reliability (ICC 0.4 to 0.7) included hip FPPA during the lateral step-down (ICC = 0.527, MDC = 10.71°), knee FPPA during the single-leg landing (ICC = 0.614, MDC = 11.90°), and trunk lean angle during the single-leg squat (ICC = 0.673, MDC = 6.97°). These results may suggest that measurements of hip FPPA, particularly during single-leg squat and lateral-step down tasks, are less reliable and less sensitive for detecting meaningful changes [13,14]. In contrast, several measures demonstrated excellent reliability (ICC > 0.9), including knee FPPA during the forward step-down (ICC = 0.941, MDC = 4.61°) and trunk lean angle during the single-leg landing (ICC = 0.947, MDC = 5.06°), indicating reliability across sessions and improved sensitivity for detecting meaningful changes [13,14].

Across the five tasks, the single-leg squat task demonstrated the lowest ICCs and the highest SEM and MDC values for trunk and lower extremity measures compared with the other tasks, suggesting increased neuromuscular demands in individuals with PFP. This reduced reliability in FPPAs may reflect underlying deficits in hip muscle function, particularly the abductors and external rotators [2]. Consistent with previous research, the single-leg squat has been identified as an important criterion for diagnosing PFP and may also predict risk for the condition, highlighting the clinical relevance of this task [15]. Based on our data, it is important to recognize that greater changes in the single-leg squat may be required to reflect clinically meaningful improvements. While these results provide useful preliminary benchmarks, the small sample size warrants cautious interpretations, as reliability estimates are less stable [14]. Accordingly, these values should be applied judiciously in both clinical and research contexts.

The dynamic valgus index demonstrated generally good to excellent reliability across tasks, with ICCs ranging from 0.775 to 0.906. However, SEM and MDC values were higher than those of isolated joint measures, likely reflecting the cumulative measurement error associated with combining hip and knee motions [16]. Scholtes and Salsich [16] reported ICCs and SEMs for females with PFP during the single-leg squat task, demonstrating an ICC of 0.74, which is comparable to the ICC observed in our study for the same task. However, they reported a larger SEM (8.63°) than that observed in our work. This difference may be related to several factors, including differences in study population, as their study included 20 females, whereas our sample consisted of 4 females and 6 males. Previous research has shown that females with PFP tend to demonstrate greater knee abduction and hip adduction during single-limb activities compared to males [17]. To our knowledge, this is the first study to report MDC values for the dynamic valgus index across multiple single-limb weight-bearing tasks in individuals with PFP, providing novel reference values for future research and clinical interpretation.

In our study, several ICC, SEM, and MDC values for FPPAs (e.g., knee FPPA during the single-leg squat and hip FPPA during the single-leg landing) were comparable to ranges previously reported in pain-free individuals [4,9,10,18]. However, individuals with PFP in our study demonstrated higher SEM and MDC values for other variables, including hip FPPA, knee FPPA, and/or trunk lean angles across the single-leg squat, single-leg landing, and lateral step-down tasks. Specifically, lower ICCs and higher SEM and MDC values were observed for hip FPPA, knee FPPA, and lateral trunk lean during the lateral step-down task [4,19,20]; knee FPPA during the single-leg landing task [4,10]; and hip FPPA and lateral trunk lean during the single-leg squat task [4,21]. As a pain-free control group was not included under the same testing protocol, these comparisons should be interpreted as contextual references rather than direct evidence of differences between individuals with and without PFP.

Time between testing sessions is an important methodological consideration in reliability studies because it can influence reliability estimates. Shorter intervals may inflate reliability due to recall, familiarization, or motor learning, whereas longer intervals may reduce reliability if a true change in movement patterns occurs over time. Prior biomechanical reliability studies [4,10,22,23] have used retest intervals ranging from five days to two weeks depending on the purpose of the study and the likelihood of clinical change. In the present study, a minimum 14-day interval was selected to reduce potential short-term learning effects while maintaining relative clinical stability in individuals with PFP. In this study, pain levels were comparable between testing sessions, suggesting that no substantial symptom changes occurred that may influence movement patterns. Therefore, the reliability values reported in our study are considered representative of measurement consistency across a clinically meaningful reassessment period.

The data reported in this study should be interpreted with caution, taking several methodological factors into consideration. First, the relatively small sample size may influence the stability of ICC estimates and the width of confidence intervals. In addition, as our study included participants with observable knee valgus during initial screening, the findings may have limited generalizability to individuals who do not exhibit this movement pattern. Future larger-scale studies including individuals with PFP both with and without knee valgus during weight-bearing tasks are essential.

## 5. Conclusions

In conclusion, we observed the lowest ICCs for hip FPPA, particularly during the single-leg squat and lateral step-down tasks. Of the five tasks tested, the single-leg squat appeared to be the most challenging, exhibiting the lowest ICCs and the highest SEM and MDC values across all four outcome measures (trunk lean angle, knee and hip FPPAs, and dynamic valgus index). The dynamic valgus index consistently showed larger SEM and MDC values than the isolated hip or knee FPPAs, likely reflecting compounded measurement errors across multiple segments. These findings provide preliminary benchmarks for interpreting measurement error and identifying meaningful change in individuals with PFP.

## Figures and Tables

**Table 1:** Frontal plane angles of the lower extremities and trunk as well as their reliability indices (ICC, SEM, MDC), across the five tasks in two testing days in participants with patellofemoral pain.

Task	Variables	Day1 Mean (SD)	Day2 Mean (SD)	ICC (95% CI)	SEM Absolute ♦ (Relative) ♦♦	MDC Absolute ♦ (Relative) ♦♦
Single-leg squat	Hip FPPA	9.48 (2.78)	9.79 (5.09)	0.396 (−2.015–0.857)	3.10 (0.32)	8.61 (0.89)
	Knee FPPA	0.85 (5.16)	2.33 (7.33)	0.894 (0.602–0.973)	2.02 (1.27)	5.61 (3.53)
	Dynamic valgus index	10.33 (7.23)	12.12 (11.87)	0.775 (0.093–0.944)	4.56 (0.41)	12.64 (1.13)
	Trunk lean angle	12.58 (4.67)	11.80 (4.33)	0.673 (−0.390–0.920)	2.52 (0.21)	6.97 (0.57)
Single-leg landing	Hip FPPA	10.09 (7.09)	7.52 (7.24)	0.876 (0.500–0.969)	2.50 (0.28)	6.93 (0.79)
	Knee FPPA	8.64 (5.95)	6.83 (7.97)	0.614 (−0.571–0.904)	4.29 (0.56)	11.90 (1.54)
	Dynamic valgus index	18.73 (11.76)	14.35 (14.56)	0.796 (0.252–0.948)	5.91 (0.36)	16.37 (1.00)
	Trunk lean angle	13.06 (8.67)	11.22 (7.49)	0.947 (0.778–0.987)	1.83 (0.15)	5.06 (0.42)
Single-leg hop	Hip FPPA	8.74 (6.47)	6.77 (6.66)	0.931 (0.670–0.984)	1.70 (0.22)	4.71 (0.61)
	Knee FPPA	2.85 (7.14)	4.25 (6.84)	0.772 (0.089–0.943)	3.27 (0.92)	9.05 (2.55)
	Dynamic valgus index	11.87 (12.96)	11.01 (12.67)	0.906 (0.616–0.977)	3.83 (0.33)	10.61 (0.93)
	Trunk lean angle	10.51 (6.42)	9.93 (7.34)	0.895 (0.573–0.974)	2.18 (0.21)	6.04 (0.59)
Forward step-down	Hip FPPA	18.22 (4.64)	18.66 (6.37)	0.771 (0.015–0.944)	2.60 (0.14)	7.20 (0.39)
	Knee FPPA	0.78 (8.95)	0.96 (12.52)	0.941 (0.758–0.985)	1.66 (0.47)	4.61 (1.30)
	Dynamic valgus index	39.92 (11.65)	45.69 (17.96)	0.883 (0.515–0.971)	4.64 (0.24)	12.86 (0.67)
	Trunk lean angle	11.54 (4.75)	11.24 (6.11)	0.847 (0.358–0.962)	2.09 (0.18)	5.78 (0.51)
Lateral step-down	Hip FPPA	24.57 (4.53)	26.86 (6.57)	0.527 (−0.734–0.880)	3.87 (0.15)	10.71 (0.42)
	Knee FPPA	15.36 (10.22)	18.84 (12.73)	0.927 (0.665–0.983)	3.07 (0.18)	8.52 (0.50)
	Dynamic valgus index	19.62 (17.27)	19.01 (9.49)	0.861 (0.446–0.965)	5.60 (0.13)	15.53 (0.36)
	Trunk lean angle	14.84 (6.00)	16.02 (5.91)	0.805 (0.446–0.965)	2.57 (0.17)	7.13 (0.46)

**♦** in degrees, **♦♦** ratio. Abbreviations: FPPA, frontal plane projection angle; SD, standard deviation. Abbreviations: FPPA, frontal plane projection angle; ICC, intraclass correlation coefficient; CI, confidence interval; SEM, standard error of measurement; MDC, minimal detectable change.

## Data Availability

The summary of the data and results relevant to this work is included in the article. The authors declare that the research was conducted in the absence of any commercial or financial relationships that could be construed as potential competing interests.

## Abbreviations

The following abbreviations are used in this manuscript:

PFP	patellofemoral pain
FPPA	frontal plane projection angle
ICC	intraclass correlation coefficient
SEM	standard error of measurement
MDC	minimal detectable change
SD	standard deviation

## References

[1] Ho K.Y., Wallace C., Aquino J., Broadwell B., Whimple M., Liang J.N. (2024). Exploring the use of bimodal transcranial direct current stimulation to enhance movement in individuals with patellofemoral pain-A sham-controlled double blinded pilot study. Front. Hum. Neurosci..

[2] Graci V., Salsich G.B. (2015). Trunk and lower extremity segment kinematics and their relationship to pain following movement instruction during a single-leg squat in females with dynamic knee valgus and patellofemoral pain. J. Sci. Med. Sport.

[3] Neal B.S., Barton C.J., Gallie R., O’Halloran P., Morrissey D. (2016). Runners with patellofemoral pain have altered biomechanics which targeted interventions can modify: A systematic review and meta-analysis. Gait Posture.

[4] Werner D.M., Di Stasi S., Lewis C.L., Barrios J.A. (2019). Test-retest reliability and minimum detectable change for various frontal plane projection angles during dynamic tasks. Phys. Ther. Sport.

[5] Weir J.P. (2005). Quantifying test-retest reliability using the intraclass correlation coefficient and the SEM. J. Strength Cond. Res..

[6] Preatoni E., Stokes K.A., England M.E., Trewartha G. (2013). The influence of playing level on the biomechanical demands experienced by rugby union forwards during machine scrummaging. Scand. J. Med. Sci. Sports..

[7] Cunningham T.J., Mullineaux D.R., Noehren B., Shapiro R., Uhl T.L. (2014). Coupling angle variability in healthy and patellofemoral pain runners. Clin. Biomech..

[8] Lopes Ferreira C., Barroso F.O., Torricelli D., Pons J.L., Politti F., Lucareli P.R.G. (2020). Women with patellofemoral pain show altered motor coordination during lateral step down. J. Biomech..

[9] Tate J., True H., Dale B., Baker C. (2015). Expert versus novice interrater and intrarater reliability of the frontal plane projection angle during a single-leg squat. Int. J. Athl. Ther. Train..

[10] Herrington L., Alenezi F., Alzhrani M., Alrayani H., Jones R. (2017). The reliability and criterion validity of 2D video assessment of single leg squat and hop landing. J. Electromyogr. Kinesiol..

[11] Marx R.G., Menezes A., Horovitz L., Jones E.C., Warren R.F. (2003). A comparison of two time intervals for test-retest reliability of health status instruments. J. Clin. Epidemiol..

[12] Koo T.K., Li M.Y. (2016). A guideline of selecting and reporting intraclass correlation coefficients for reliability research. J. Chiropr. Med..

[13] Atkinson G., Nevill A.M. (1998). Statistical methods for assessing measurement error (reliability) in variables relevant to sports medicine. Sports Med..

[14] Hopkins W.G. (2000). Measures of reliability in sports medicine and science. Sports Med..

[15] Willy R.W., Hoglund L.T., Barton C.J., Bolgla L.A., Scalzitti D.A., Logerstedt D.S., Lynch A.D., Snyder-Mackler L., McDonough C.M., Altman R. (2019). Patellofemoral pain: Clinical practice guidelines linked to the international classification of functioning, disability and health from the academy of orthopaedic physical therapy of the American physical therapy association. J. Orthop. Sports Phys. Ther..

[16] Scholtes S.A., Salsich G.B. (2017). A dynamic Valgus index that combines hip and knee angles: Assessment of utility in females with patellofemoral pain. Int. J. Sports Phys. Ther..

[17] Nakagawa T.H., Moriya É.T.U., Maciel C.D., Serrão A.F.V. (2012). Frontal plane biomechanics in males and females with and without patellofemoral pain. Med. Sci. Sports Exerc..

[18] Gwynne C.R., Curran S.A. (2014). Quantifying frontal plane knee motion during single limb squats: Reliability and validity of 2-dimensional measures. Int. J. Sports Phys. Ther..

[19] Jones D., Tillman S.M., Tofte K., Mizner R.L., Greenberg S., Moser M.W., Chmielewski T.L. (2014). Observational ratings of frontal plane knee position are related to the frontal plane projection angle but not the knee abduction angle during a step-down task. J. Orthop. Sports Phys. Ther..

[20] Simon M., Parizek C., Earl-Boehm J.E., Bazett-Jones D.M. (2018). Quantitative and qualitative assessment of frontal plane knee motion in males and females: A reliability and validity study. Knee.

[21] Doozan M., Bazett-Jones D.M., Glaviano N.R. (2021). Novice versus expert intertester reliability of two-dimensional squatting kinematics in females with and without patellofemoral pain. Int. J. Athl. Ther. Train..

[22] Munro A., Herrington L., Carolan M. (2012). Reliability of 2-dimensional video assessment of frontal-plane dynamic knee valgus during common athletic screening tasks. J. Sport Rehabil..

[23] Fortin C., Nadeau S., Labelle H. (2008). Inter-trial and test-retest reliability of kinematic and kinetic gait parameters among subjects with adolescent idiopathic scoliosis. Eur. Spine J..

